# Descriptive Epidemiology of Serious Work-Related Injuries in British Columbia, Canada

**DOI:** 10.1371/journal.pone.0038750

**Published:** 2012-06-19

**Authors:** Jonathan Fan, Christopher B. McLeod, Mieke Koehoorn

**Affiliations:** 1 School of Population and Public Health, University of British Columbia, Vancouver, British Columbia, Canada; 2 College for Interdisciplinary Studies, University of British Columbia, Vancouver, British Columbia, Canada; 3 Centre for Health Services and Policy Research, School of Population and Public Health, University of British Columbia, Vancouver, British Columbia, Canada; Chancellor College, University of Malawi, Malawi

## Abstract

**Objective:**

This study examined the rates and distribution of serious work-related injuries by demographic, work and injury characteristics in British Columbia, Canada from 2002–2008, using population-based data.

**Methods:**

Claims for workers with a serious injury were extracted from workers’ compensation data. Serious injuries were defined by long duration, high cost, serious medical diagnosis, or fatality. Workforce estimates were used to calculate stratum-specific rates. Rate-ratios (RR) and 95% CIs were calculated using negative binomial regression for the comparison of rates, adjusting for gender, age and occupation.

**Results:**

Women had a lower overall serious injury rate compared to men (RR: 0.93, 95% CI: 0.87–0.99). The 35–44 age group had the highest overall rate compared to the youngest age group. The rate for severe strains/sprains was similarly high for men and women in the 35–44 age group, although there was a differential pattern by gender for other injury types: the rate of fracture was similar across age groups for men, but increased with age for women (RR: 2.7, 95% CI: 2.2–3.3); and the rate of severe falls increased with age for men and women, with a larger three-fold increase for older women (men: RR: 1.8, 95% CI: 1.7–2.1; women: RR: 3.2, 95% CI: 2.7–3.7).

**Conclusions:**

The risk of serious injuries is higher among specific age groups with different patterns emerging for men and women. Variations persisted within similar injury types and occupation groups in our adjusted models. These results provide evidence for the burden of serious injuries and a basis for future analytic research. Given projected demographic shifts and increasing workforce participation of older workers, intervention programs should be carefully implemented with consideration to demographic groups at risk for serious injuries in the workplace.

## Introduction

Occupational injuries in high-income countries have been declining over the past two decades [1]–[3]. In Canada, for example, the number of work-related injury claims fell from 621 000 in 1989 to 260 000 in 2009, a reduction of 58% [1]. The United States has also experienced declines in the number and rate of work-related injuries and illnesses, including a 22% decrease in the rate of non-fatal claims among private industries since 2003 [3]. While there has been a reduction in overall workplace injuries, serious or severe injuries have not decreased commensurately in some jurisdictions [4]. Serious injuries result in longer disability, higher claim costs, fatalities, and/or severe medical diagnoses (such as fractures, dislocations, or amputations); and impart a significant and disproportionate burden on compensation and health systems [5]. In the United States, the direct cost of the most disabling workplace injuries amounted to over $53 billion USD, representing 71% of the total compensation cost burden in 2008 [6]. In the United Kingdom, slips, trips and falls (the most common cause of major workplace injury in the UK) accounted for approximately $1.3 billion USD in total costs to society [7]. In the Canadian province of British Columbia, serious injuries comprised one-third of overall claims, yet accounted for 75% of the disability days paid and 85% of the associated claim costs in 2009 [5].

A number of studies have specifically examined serious work-related injuries, with the majority showing that older workers suffer from a greater number of severe or fatal injuries when compared to their younger counterparts, despite an overall higher rate of injury among younger workers [8]–[15]. Although this pattern has been well documented, there is a lack of surveillance or descriptive epidemiology on the correlates of these injuries, and less evidence on the risk of severe injuries within specific occupation groups [14], [16]. Using population-based claims data and labour force survey data, the objectives of this study were to estimate the rates and distribution of serious occupational injuries in British Columbia, Canada by demographic, work and injury characteristics for the period of 2002 to 2008.

## Methods

We used administrative claims data maintained by WorkSafeBC (the Workers’ Compensation Board of British Columbia). WorkSafeBC is an independent statutory agency governed by a Board of Directors appointed by the provincial government of British Columbia (BC) [4]. This compensation system administers the provincial Workers’ Compensation Act, which gives legal authority to set and enforce occupational health and safety standards and policies. WorkSafeBC has a prevention and rehabilitation mandate, and administers claims for workers who are adjudicated to have been injured while at work. An estimated 93% of BC’s provincial workforce is covered under this no-fault compensation system, which is funded primarily through insurance premiums paid by registered employers. WorkSafeBC allows for a few exemptions from coverage (or is not responsible for coverage) and as such under-represents the self-employed and small workplaces (e.g., 1–2 workers), and some federal workers (e.g., federal police, military). Data available from the WorkSafeBC claims database includes information on the injuries accepted for compensation; data used for this analysis include demographic variables (age, gender), injury characteristics (diagnosis, cause, source, date of injury), claim outcome (health care only, short- or long-term disability, fatality), and occupation at time of injury.

We extracted claims for workers (aged 15 years and older) with a serious injury in BC, between 2002 and 2008. The serious injury criteria included individuals with an accepted short-term (at-least 1 day of time loss) or long-term disability claim; and at-least one of the following: 1) long duration (≥28 days of wage loss); 2) high claim costs (equivalent to 28 days of wage loss); 3) serious medical diagnosis (e.g., fractures, dislocations, severe strains/sprains, open wounds, amputations); or, 4) fatality. These criteria were developed by WorkSafeBC to provide a measure of serious injury related to duration of disability, claim cost, and injury type [4]. The medical diagnosis codes for serious injury encompass a wide range of diagnoses, but mainly focus on injuries from ICD-9 Chapter 17: Injury and Poisoning, including fractures, strains and sprains, open wounds, contusions, dislocations, internal injuries, amputations, crush injuries, and burns.

Workforce estimates from Statistics Canada’s Labour Force Survey [17] were used as the denominator to calculate claim rates of serious injury per 1 000 workers in BC, for each year of the study period, by gender, age (10-year groupings), and occupation (Statistics Canada’s Standard Occupational Classification). Rates were calculated for overall serious injury and for specific injury types defined by diagnosis and cause. Confidence intervals for the rates were calculated at the 95%-level assuming Poisson-distributed counts.

Negative binomial regression was used to calculate rate-ratios (RR) and 95% confidence intervals (95% CI) for the comparison of claim rates. A likelihood-ratio test for over-dispersion indicated the use of a negative binomial regression model rather than a Poisson regression model. Rate-ratios were calculated using all claims data over the study period, and adjusted for age, occupation, and year of injury. Rate-ratios were additionally stratified by gender. Statistical analyses were completed using Stata IC/10.1 [18].

### Ethics Statement

The Behavioural Research Ethics Board of the University of British Columbia (UBC) reviewed and approved the research protocol. This study was based on secondary usage of existing administrative data on workers’ compensation claims controlled by WorkSafeBC. Access to this data was provided under a data access agreement between UBC and WorkSafeBC governing the privacy and confidentiality conditions for use of the data for research purposes. Researchers with approved access to data collected by a designated public body such as WorkSafeBC are not required to seek individual consent for the use of administrative data for research and statistical purposes.

## Results

A total of 127 808 serious injury claims were identified in BC from 2002 to 2008 (**Table 1**). Men accounted for 2 out of 3 of these claims. The overall claim rate was relatively stable over the study period, ranging from 7.9 to 8.4 per 1 000 workers per year (adjusted RR: 1.08, 95% CI: 0.96–1.22; for 2008 compared to 2002). The majority of these claims matched the long disability-duration criteria (92%) for a serious injury, while 25% of the claims resulted in serious medical diagnoses. Less than 1% of serious injury claims resulted in a fatality over the 2002 to 2008 period (n = 532). In 2008, the most recent year of data available, there were 19 687 serious injury claims, for a rate of 8.2 per 1000 workers.

**Table 1:** Number and rate of serious injury claims in British Columbia, Canada, 2002 to 2008, by demographic and work characteristics.

				Unadjusted^a^		Adjusted^b^	
	Injuries	Labour force (1000)	Rate per 1000	Rate-ratio	95% CI	Rate-ratio	95% CI
**Year of injury**							
2002	17499	2083	8.4	1	–	1	–
2003	16859	2129	7.9	0.94	0.92–0.96	0.95	0.84–1.07
2004	17525	2171	8.1	0.96	0.94–0.98	0.94	0.84–1.06
2005	18322	2217	8.3	0.98	0.96–1.00	1.00	0.89–1.13
2006	18630	2270	8.2	0.98	0.96–1.00	1.00	0.89–1.13
2007	19286	2336	8.3	0.98	0.96–1.00	1.01	0.90–1.14
2008	19687	2394	8.2	0.98	0.96–1.00	1.08	0.96–1.22
**Gender**							
Men	86926	8295	10.5	1	–	1	–
Women	40882	7305	5.6	0.53	0.51–0.56	0.93	0.87–0.99
**Age (years)**							
15–24	14673	2496	5.9	1	–	1	–
25–34	25717	3263	7.9	1.34	1.29–1.40	1.19	1.07–1.33
35–44	36321	3861	9.4	1.60	1.54–1.67	1.37	1.23–1.52
45–54	33866	3766	9.0	1.53	1.47–1.59	1.29	1.16–1.44
55–64	15763	1912	8.2	1.40	1.34–1.46	1.21	1.08–1.35
65+	1468	303	4.9	0.82	0.77–0.88	0.67	0.59–0.77
**Occupation**							
Management	2544	1404	1.8	1	–	1	–
Admin, professional	10772	5425	2.0	1.10	1.03–1.17	1.03	0.91–1.17
Health	11706	881	13.3	7.34	6.88–7.82	4.80	4.21–5.47
Sales, service	26770	4164	6.4	3.55	3.34–3.78	3.51	3.09–3.98
Trades, transport, construction	52603	2460	21.4	11.82	11.11–12.57	9.99	8.80–11.34
Natural resources	7942	546	14.5	8.04	7.53–8.58	6.17	5.43–7.02
Manufacturing, utilities	15471	721	21.5	11.86	11.14–12.63	9.65	8.49–10.97
**Overall (2002 to 2008)**	127808	15,600	8.2	–	–	–	–

a Rate-ratios and 95% confidence intervals obtained via negative binomial regression.

b Rate-ratios adjusted for gender, age, occupation and year of injury.

Over the study period, women had lower rates of serious injury compared to men (unadjusted RR: 0.53, 95% CI: 0.51–0.56), although this difference was reduced when controlling for occupation (adjusted RR: 0.93, 95% CI: 0.87–0.99). The 35–44 age group had the highest overall rate of serious injury compared to the youngest age group (15–24 years), with an increase of nearly 40% in the adjusted models (adjusted RR: 1.37, 95% CI: 1.23–1.52). **(** **Table 1** **)**.

Similar age-related patterns were also observed when examining rates separately for men and women, with the rate of serious injury highest among the 35–44 age groups in the adjusted models. For men, the rate for the 35–44 age group was 39% higher compared to the 15–24 age group (adjusted RR: 1.39; 95% CI: 1.27–1.53); and for women, the rate was 44% higher (adjusted RR: 1.44, 95% CI: 1.27–1.65). **(** **Table 2** **)**.

**Table 2:** Number and rate of serious injury claims in British Columbia, Canada, 2002 to 2008, by demographic and work characteristics, and stratified by gender.

	Men				Women			
	Injuries	Rate per 1000	Adjustedrate-ratio^a^	95% CI	Injuries	Rate per 1000	Adjustedrate-ratio^a^	95% CI
**Year of injury**								
2002	12100	10.9	1	–	5399	5.5	1	–
2003	11795	10.4	0.95	0.86–1.05	5064	5.1	0.95	0.82–1.10
2004	12212	10.6	0.93	0.84–1.03	5313	5.2	0.96	0.83–1.11
2005	12543	10.6	0.97	0.88–1.08	5779	5.6	1.03	0.89–1.18
2006	12621	10.5	0.95	0.86–1.05	6009	5.6	1.06	0.92–1.22
2007	12761	10.2	0.94	0.85–1.03	6525	6.0	1.10	0.95–1.27
2008	12894	10.1	0.92	0.84–1.02	6793	6.1	1.25	1.08–1.44
**Age (years)**								
15–24	10993	8.7	1	–	3680	3.0	1	–
25–34	18708	10.8	1.27	1.16–1.39	7009	4.6	1.22	1.07–1.39
35–44	24428	12.0	1.39	1.27–1.53	11893	6.5	1.44	1.27–1.65
45–54	21199	10.7	1.26	1.15–1.38	12667	7.1	1.42	1.25–1.62
55–64	10417	9.6	1.16	1.05–1.27	5346	6.5	1.33	1.16–1.53
65+	1181	5.9	0.76	0.68–0.85	287	2.8	0.61	0.51–0.73
**Occupation**								
Management	1562	1.7	1	–	982	1.9	1	–
Admin, professional	5464	2.5	1.41	1.26–1.58	5308	1.7	0.79	0.68–0.93
Health	952	5.3	2.80	2.46–3.18	10754	15.4	6.54	5.60–7.63
Sales, service	10123	5.8	3.52	3.16–3.93	16647	6.9	3.67	3.15–4.27
Trades, transport, construction	49553	21.5	12.50	11.22–13.94	3050	19.1	8.45	7.22–9.89
Natural resources	6750	16.6	9.02	8.07–10.07	1192	8.5	4.15	3.53–4.89
Manufacturing, utilities	12522	23.2	13.09	11.72–14.62	2949	16.3	7.45	6.36–8.73
**Overall (2002 to 2008)**	86926	10.5	–	–	40882	5.6	–	–

a Rate-ratios stratified by gender, and adjusted for age, occupation and year of injury.

### Occupation

Over the study period, the occupations with the highest serious injury rate were manufacturing & utilities (21.5 per 1 000); trades, transport & construction (21.4); natural-resource occupations (14.5); and health (13.3). The lowest rates were observed in management (1.8); and administration and professional occupations (2.0). (**Table 1**).

Rates were higher for men compared to women across high-risk occupations, with the exception of health, where women had three times the rate of men (men: 5.3; women: 15.4). For men, the highest rates of serious injury occurred in manufacturing & utilities; trades, transport & construction; and natural-resource occupations. For women, the highest rates occurred in trades, transport & construction; manufacturing & utilities; and health occupations. (**Table 2**).

### Serious Injury Characteristics

#### Injury diagnosis

Table 3 provides the distribution and rates of serious injury claims for the most common injury diagnoses in the study sample. The overall rate of sprains and strains was 4.1 per 1 000 workers. The 35–44 age group had the highest rate compared to the youngest age group (15–24 years), and this pattern was consistent for both men and women in the adjusted models. For men, the rate was 67% higher for the 35–44 age group versus the 15–24 age group (adjusted RR: 1.67, 95% CI: 1.50–1.86); and for women, the rate was 75% higher (adjusted RR: 1.75, 95% CI: 1.52–2.00).

**Table 3:** Number and rate of serious injuries for the most frequent diagnoses, by gender and age group, 2002 to 2008.

	Men				Women				Total	
	Injuries	Rate per 1000	Adjustedrate-ratio^a^	95% CI	Injuries	Rate per 1000	Adjusted rate-ratio^a^	95% CI	Injuries	Rate per 1000
**Strains and sprains**										
15–24	4208	3.3	1	–	2059	1.7	1	–	6267	2.5
25–34	8494	4.9	1.47	1.32–1.64	4617	3.0	1.43	1.25–1.65	13111	4.0
35–44	11331	5.6	1.67	1.50–1.86	7889	4.3	1.75	1.52–2.00	19220	5.0
45–54	9466	4.8	1.43	1.29–1.60	8140	4.5	1.64	1.43–1.88	17606	4.7
55–64	4533	4.2	1.29	1.16–1.45	2957	3.6	1.28	1.11–1.48	7490	3.9
65+	383	1.9	0.63	0.54–0.73	91	0.9	0.38	0.29–0.49	474	1.6
	38415	4.6	–	–	25753	3.5	–	–	64168	4.1
**Fractures**										
15–24	2830	2.2	1	–	514	0.4	1	–	3344	1.3
25–34	4200	2.4	1.18	1.07–1.30	590	0.4	0.85	0.69–1.05	4790	1.5
35–44	5271	2.6	1.21	1.10–1.33	981	0.5	1.07	0.88–1.31	6252	1.6
45–54	4840	2.5	1.15	1.05–1.26	1429	0.8	1.55	1.27–1.88	6269	1.7
55–64	2566	2.4	1.17	1.06–1.29	1205	1.5	2.71	2.22–3.31	3771	2.0
65+	389	2.0	1.06	0.92–1.22	135	1.3	2.18	1.68–2.83	524	1.7
	20096	2.4	–	–	4854	0.7	–	–	24950	1.6

a Rate-ratios stratified by gender, and adjusted for age, occupation and year of injury.

The overall rate of fractures was 1.6 per 1 000 workers. The rate increased steadily with age, reaching 2.0 per 1 000 among the 55–64 age group (although the rate dropped slightly in the oldest age group, 65+ years). However, there was a differential pattern across age groups when examined separately for men and women, with men having less variation in rates (adjusted RR: 1.17, 95% CI: 1.06–1.29; for 55–64 versus 15–24 years) compared to women (adjusted RR: 2.71, 95% CI: 2.22–3.31; for 55–64 versus 15–24 years).

#### Injury cause

Table 4 provides the distribution and rates of serious injury claims for the most common causes of injury in the study sample. The overall rate of overexertion/repetitive motion was 2.3 per 1 000 workers. The 35–44 age group had the highest rate compared to the youngest age group (15–24 years), and this pattern was consistent for both men and women in the adjusted models. For men, the rate was 83% higher for the 35–44 age group versus the 15–24 age group (adjusted RR: 1.83, 95% CI: 1.65–2.03); and for women, the difference was two-fold higher (adjusted RR: 2.04, 95% CI: 1.76–2.36).

**Table 4:** Number and rate of serious injuries for the most frequent causes of injury, by gender and age group, 2002 to 2008.

	Men				Women				Total	
	Injuries	Rate per 1000	Adjusted rate-ratio^a^	95% CI	Injuries	Rate per 1000	Adjusted rate-ratio^a^	95% CI	Injuries	Rate per 1000
**Overexertion, repetitive motion**										
15–24	2273	1.8	1	–	1126	0.9	1	–	3399	1.4
25–34	4677	2.7	1.57	1.41–1.74	2694	1.8	1.58	1.36–1.83	7371	2.3
35–44	6337	3.1	1.83	1.65–2.03	4697	2.6	2.04	1.76–2.36	11034	2.9
45–54	5072	2.6	1.52	1.37–1.69	4681	2.6	1.87	1.61–2.17	9753	2.6
55–64	2281	2.1	1.31	1.18–1.47	1520	1.8	1.32	1.12–1.54	3801	2.0
65+	166	0.8	0.57	0.48–0.69	46	0.4	0.41	0.30–0.57	212	0.7
	20806	2.5	–	–	14764	2.0	–	–	35570	2.3
**Falls**										
15–24	2294	1.8	1	–	864	0.7	1	–	3158	1.3
25–34	4253	2.5	1.44	1.30–1.60	1462	1.0	1.42	1.21–1.67	5715	1.8
35–44	6049	3.0	1.69	1.53–1.87	2489	1.4	1.85	1.58–2.17	8538	2.2
45–54	5914	3.0	1.73	1.56–1.91	3296	1.8	2.36	2.02–2.76	9210	2.4
55–64	3299	3.0	1.84	1.66–2.05	2070	2.5	3.18	2.71–3.73	5369	2.8
65+	488	2.5	1.60	1.39–1.83	178	1.7	2.02	1.62–2.52	666	2.2
	22297	2.7	–	–	10359	1.4	–	–	32656	2.1
**Contact with objects**										
15–24	4211	3.3	1	–	726	0.6	1	–	4937	2.0
25–34	5342	3.1	1.02	0.94–1.11	1016	0.7	1.12	0.94–1.32	6358	1.9
35–44	6086	3.0	0.99	0.91–1.07	1540	0.8	1.29	1.09–1.52	7626	2.0
45–54	4990	2.5	0.84	0.77–0.91	1445	0.8	1.17	0.99–1.39	6435	1.7
55–64	2253	2.1	0.71	0.65–0.78	550	0.7	0.99	0.83–1.19	2803	1.5
65+	236	1.2	0.47	0.40–0.55	21	0.2	0.30	0.19–0.47	257	0.8
	23118	2.8	–	–	5298	0.7	–	–	28416	1.8

a Rate-ratios stratified by gender, and adjusted for age, occupation and year of injury.

The overall rate of fall injuries was 2.1 per 1 000 workers. The rate increased steadily with age, reaching 2.8 among the 55–64 age group (although the rate dropped off in the oldest age group, 65+ years). For men, the rate was nearly two-times higher for the 55–64 age group versus the 15–24 age group (adjusted RR: 1.84, 95% CI: 1.66–2.05); and for women, the rate was over three-times higher for the same age group (adjusted RR: 3.18, 95% CI: 2.71–3.73).

Conversely, the rate of injuries due to contact with objects (including struck by/against, caught in) declined with age, reaching 0.8 per 1 000 among the 65+ age group. However, when stratified by gender, the rate of contact injuries for women peaked in the 35–44 age group compared to the youngest age group (adjusted RR: 1.29, 95% CI: 1.09–1.52).

## Discussion

This study identified several groups at higher risk for serious injury using population-based workers’ compensation data. Men had higher rates compared to women overall, and the 35–44 age group had the highest overall serious injury rate in the adjusted models. The rate for strains/sprains and severe overexertion/repetitive motion injuries was also high in this age group, although older age groups had significantly higher rates of other specific injury types (such as fractures and severe falls) in the adjusted models. Moreover, the association between age and serious injuries varied by gender, with distinct patterns emerging for men and women.

### Demographic Characteristics

In our study, the 35–44 age group had higher overall rates of serious injury compared to the youngest age group, with an increase of nearly 40% in the adjusted models. The risk of specific diagnoses and causes of serious injury was also higher among certain age groups. For example, the middle age-groups (e.g., 35–44 years) had higher rates of strains & sprains and severe overexertion/repetitive motion injuries; whereas older workers (e.g., 55–64 years) had two- to three-times the rate of fractures and severe falls compared to the youngest age group. Conversely, older workers had lower rates of contact injuries compared to the youngest age group.

The above findings are consistent with a number of previous studies [8]–[15], which have shown that older workers suffer from a greater number of severe or fatal injuries when compared to their younger counterparts, despite an overall higher rate of injury among younger workers. Moreover, differential age-patterns across injury subsets have been reported in previous studies using occupational injury data [19]. For example, a 2011 report by the Centers for Disease Control and Prevention [19] examined nonfatal occupational injuries using United States national-level survey data, and found that older workers had higher rates of falls and fractures compared to younger workers, with rates steadily increasing with age; whereas younger workers had higher rates of contact injuries compared to older workers. This is consistent with the age-related increase in fractures and severe fall injuries; and the age-related decline in injuries due to contact with objects in our study. Another study by Choi et al. [20] examined workers’ compensation claims data from Ontario, Canada, and found that workers aged 30 to 59 years were more likely to have strain & sprain injuries; whereas workers aged less than 30 years and older than 59 years had decreased risk. Similarly, in our study, we found high rates of strains & sprains and severe overexertion/repetitive motion injuries among workers in the middle of the age distribution (e.g., 35–44 years), with the lowest rates observed among the youngest (15–24 years) and oldest (65+ years) age groups.

We also found distinct patterns of risk when examining serious injuries separately for men and women. For example, the rate of fracture had less variation across age groups for men, whereas the rate for women increased steadily with age. There was also a greater association between age and severe fall-related injuries for women, with older women having substantially higher rates compared to younger women (and exceeding the rate for men for falls on the same level; data not shown). Conversely, the rate of contact injuries for men declined consistently with increasing age, but for women, the rates peaked in the 35–44 age group.

These findings suggest that women in the 35–44 and 55–64 age groups are at increased risk for certain diagnoses and causes of serious injuries. This is supported by previous studies on fractures, which suggest that the risk of fall-related fracture increases with age for women [21]–[23], with men having a different risk profile across age groups [22], [23]. Moreover, a recent study by Alamgir et al. [24] examined risk factors for serious falls among British Columbia health care workers and found that women had longer durations of disability and higher claim costs compared to men. There are a number of biological, behavioural, environmental, and socioeconomic factors that may explain the higher incidence of falls and fractures among older workers, and women in particular [25]. As an example, older women may use multiple medications that affect physical functioning and predispose them to falls; or experience muscle mass or bone mass declines at a faster rate [25].

These findings also parallel a growing public health concern among an aging population in general, with several reports highlighting the increasing burden of falls among community-dwelling older adults in the US and Canada. For example, approximately 1 in 3 adults over the age of 65 will fall each year [25], with older women experiencing a higher rate of falls and fall-related fractures compared to older men [26]. Studies using population-based data have shown significant increases in fatality rates for fall-related injuries among the older population [27]–[29]. In an occupational setting, falls, fractures and hospitalizations also primarily occur among the oldest workers [13], [19], [30]. Falls, in particular, have a considerable economic impact for both workers’ compensation and health care systems [31]. According to US data, falls accounted for the greatest proportion of workers’ compensation costs for disabling injuries, while falls on the same level experienced a 42% growth in direct costs of disabling workplace injuries over the last decade [6]. Similarly, in British Columbia, falls on the same level accounted for a substantial proportion of compensation claims over the past decade [5].

### Work Characteristics

Physical job demands may account for a large proportion of the variation in serious injury rates between men and women, and older and younger workers. A 2005 study by Breslin et al. [16] examined work-related injuries in Canada using population-based data, and found that younger men and women had elevated risk of injury requiring medical attention compared to older workers. This difference in risk, however, was substantially reduced when controlling for job characteristics such as occupation classification and physical exertion. Another study by Mitchell et al. [14] examined workers’ compensation claims from 29 states in the US, and found that the difference in risk of work-related permanent disability and fatality across age groups was reduced, although not eliminated, after controlling for occupation and industry.

In our study, men had higher overall serious injury rates compared to women, although this difference was reduced when adjusting for occupation group. While physical job demands may help to explain the discrepancy in findings across demographic variables in our study, there was still a difference in rates for men and women even within specific occupation groups. Women in health occupations, for example, had three- to four-times the rate of serious injury compared to men in the same occupations, and this difference was consistent within detailed job titles (data not shown), such as technical occupations in health (e.g., ambulance attendants, RN assistants), and professional occupations (e.g., physicians). We note that occupation group is an indirect measure of work-related exposure, and that the possibility remains for residual differences in exposures and physical demands, even within specific job titles [32]. Nevertheless, in our adjusted regression models that controlled for occupation, we still found significant differences in serious injury rates across age groups for men and women, within similar injury types and occupation groups.

### Demographic Trends

Serious injuries represent a proportion of the overall injury rate, but the burden of these injuries is concentrated among specific age groups and in high-risk occupations. The anticipated increase in the number of older workers may lead to increases in serious injuries that are age-related. For example, between 2001 and 2006, the Canadian workforce aged 55 to 64 grew faster than any other age group, representing 16.9% of the workforce, up from 14.1% in 2001 [33]. This is largely the result of an aging population coupled with an increase in workforce participation among older adults, with older women experiencing a steady increase over the past two decades [34]. The prevention of serious injuries in workplaces will have implications beyond worker safety and compensation policy that must be considered in relation to an aging workforce, with specific attention to demographic groups at risk for serious injury.

### Strengths and Limitations

The strength of this study was the use of a sample that is representative of all claims for serious injury obtained from population-based data at the individual-level; coupled with demographic, work, and injury characteristics that were used to calculate detailed rates for specific types of serious injury. Although our data characterizes the vast majority of BC’s employed workforce (93%) as covered under the provincial workers’ compensation system, some workers such as the self-employed and some federal workers are excluded and this may bias the results downward. There is also the possibility that some workplace injuries are not reported for compensation. However, our study likely captures the majority of claims since severe injuries are the most likely to be reported [35]–[37].

### Conclusions

The risk of serious injuries is higher among specific age groups with different patterns emerging for men and women. Moreover, these variations persisted within similar injury types and occupation groups in our adjusted models. These results give evidence for the burden of serious work-related injury using population-based data, and provide a basis for future analytic research. Understanding the causes and consequences of serious injuries will inform the development of effective interventions to prevent serious injuries among workers. Given projected demographic shifts and increasing workforce participation of older workers, stakeholders and public health officials should carefully implement these programs with consideration to demographic groups at-risk for certain types of serious injuries, such as strains/sprains, fractures and severe falls, in the workplace.
